# Multi-informant Implementation and Intervention Outcomes of Opioid Overdose Education and Naloxone Distribution in New York City

**DOI:** 10.1007/s43477-021-00021-4

**Published:** 2021-09-20

**Authors:** Laura Brandt, Takuya Yanagida, Aimee N. C. Campbell, Jermaine D. Jones, Marie-Therese Schultes, Suky Martinez, Sandra D. Comer

**Affiliations:** 1 Division on Substance Use Disorders, Department of Psychiatry & New York State Psychiatric Institute, Columbia University Irving Medical Center, 1051 Riverside Drive, Unit #120, New York, NY 10032 USA; 2 Department of Developmental and Educational Psychology, University of Vienna, Universitaetsstrasse 7 (NIG), 1010 Vienna, Austria; 3 Institute for Implementation Science in Healthcare, University of Zurich, Universitaetstrasse 84, 8006 Zurich, Switzerland

**Keywords:** Overdose Education and Naloxone Distribution, Fidelity, Implementation outcomes, Acceptability, Overdose knowledge, Multi-informant measures

## Abstract

Overdose Education and Naloxone Distribution (OEND) is an effective public health intervention to reduce opioid overdose fatalities (McDonald and Strang, Addiction 111:1177–1187, 2016). However, we know little about OEND implementation outcomes (i.e., indicators of implementation success), specifically the fidelity of training delivery, and how these may relate to intervention outcomes (i.e., indicators of the success or effectiveness of an intervention), such as overdose knowledge and attitudes. This study evaluated 16 OEND trainings conducted at different Opioid Overdose Prevention Programs in New York City. Trainees (*N* = 75) completed the Opioid Overdose Knowledge and Attitude Scales before and after training (intervention outcomes). Implementation outcomes were fidelity (competence and adherence of the trainer, *N* = 10; modified Fidelity Checklist) and acceptability of OEND (Acceptability of Intervention Measure), assessed from multiple perspectives (trainees, trainers, and an independent observer). Trainees’ overdose knowledge, *t*(71) = − 8.12, *p* < 0.001, 95% CI [− 6.54, − 3.96], and attitudes, *t*(65) = − 6.85, *p* < 0.001, 95% CI [− 0.59, − 0.33], improved significantly from pre- to post-training. Stepwise multiple regression models indicated that adherence of the trainer rated from the observer perspective added significantly to the prediction of changes in overdose knowledge, *F*(1, 67) = 9.81, *p* = 0.003, and explained 13% of the variance in outcome. However, fidelity measures from the perspective of trainees or trainers and acceptability of OEND were not associated with changes in trainees’ overdose knowledge or attitudes. OEND implementation outcomes and their relationship with intervention outcomes differed depending on the role of the fidelity rater in relation to the intervention. Specifically, our findings indicate that fidelity should be measured from an independent perspective (i.e., an individual who is experienced with fidelity rating but not directly involved in the intervention).

**Supplementary Information:**

The online version contains supplementary material available at 10.1007/s43477-021-00021-4.

## Introduction

### Opioid-Related Overdose Deaths and Targeted Response

Reducing fatal opioid overdoses remains a major challenge for public health. Drug overdose continues to be one of the main causes of death among people who use substances, and opioids are present in most overdose cases (Mattson et al., 2021). While the European Monitoring Center for Drugs and Drug Addiction has noted an increase in drug-related deaths since 2014 (European Monitoring Centre for Drugs & Drug Addiction, 2019, 2020a), the opioid epidemic has mainly affected North America. In 2019, nearly 50,000 opioid-related overdose deaths were reported in the United States (US) (Mattson et al., 2021).

Many opioid overdose deaths are preventable, using a comprehensive approach that includes prevention, treatment of opioid use disorder, and raising public awareness (Levine & Fraser, 2018). Given that overdoses often occur in the presence of a witness (Lagu et al., 2006; Sporer, 2003; Strang et al., 2000; Tracy et al., 2005; Wagner et al., 2015), programs have been implemented where laypersons are given brief education in recognizing the signs of opioid overdose. Overdose education typically also covers appropriate first aid including the provision of naloxone (Overdose Education and Naloxone Distribution, OEND) (Doe-Simkins et al., 2009). Naloxone is a short-acting opioid receptor antagonist effective in counteracting the respiratory depression that can lead to death during opioid overdose (White & Irvine, 1999) and has an excellent pharmacological safety profile (i.e., side effects from naloxone are rare) (Sporer et al., 1996; Wermeling, 2015; Yealy et al., 1990).

Training individuals who use substances in recognizing and managing opioid overdose improves their knowledge of overdose risk factors and symptoms, and appropriate emergency response (Heavey et al., 2018; Neale et al., 2019; Strang et al., 2008). Moreover, naloxone administration by laypersons is associated with significantly increased odds of overdose recovery compared with no naloxone administration (Giglio et al., 2015), and OEND has a measurable effect on reducing the rate of overdose deaths while having a very low rate of adverse events (Albert et al., 2011; McDonald & Strang, 2016; Tzemis et al., 2014; Walley et al., 2013).

Since 1996, OEND has become a widely used overdose harm reduction practice in the US (Centers for Disease Control and Prevention (CDC), 2012; Davis & Carr, 2017; Lambdin et al., 2020; Wheeler et al., 2015). All 50 states currently allow prescribing of naloxone to laypersons at risk of experiencing or witnessing an opioid overdose (Prescription Drug Abuse Policy System, 2019). In Europe, the implementation of OEND lags behind the US (McDonald et al., 2017)*.* National, regional or local Opioid Overdose Prevention Programs—also referred to as Take-Home Naloxone programs—have been implemented in 12 countries (European Monitoring Centre for Drugs & Drug Addiction, 2020b), and a joint WHO and United Nations Office of Drugs and Crime initiative started OEND implementation in Kazakhstan, Kyrgyzstan, Tajikistan, and Ukraine (World Health Organization, 2020). As an important step towards a wider dissemination of OEND, the WHO (World Health Organization, 2014) released guidelines recommending that naloxone should be made available to anyone likely to witness an overdose.

With increasing evidence on the benefits of OEND, wider implementation can be expected. However, there are currently no national guidelines in the US for the implementation of these programs. In the US as well as in Europe, different services have produced a range of training protocols over the years, varying in their format, content, prescribing procedures, and level of training. These variations make it difficult to determine which contents are routinely covered in trainings, and to understand how this may influence outcomes (Clark et al., 2016).

### Taking an Implementation Perspective on Overdose Prevention Efforts

Over the past two decades, research related to developing and implementing evidence-based practices has improved considerably; however, a gap exists between our knowledge of effective interventions and services being received by consumers (Fixsen et al., 2015). Innovative community-based programs (such as OEND) are not self-executing. They require an implementation perspective, i.e., an approach that views postadoption events as crucial and focuses on the actions of those who convert the intervention into practice as the key to success or failure (Petersilia, 1990). There is strong empirical support that implementation outcomes are associated with the outcomes obtained in promotion and prevention programs (Durlak & DuPre, 2008). Therefore, the collection of implementation data in addition to intervention outcome data is essential to obtain valid information about how an evidence-based practice can be optimized in “real-world” settings.

Acceptability and fidelity are among the key implementation outcomes throughout all stages of implementation—from early adoption to long-term sustainability (Proctor et al., 2011). Acceptability is often used in formative research or pilot studies as one of the leading indicators of implementation success (Bowen et al., 2009) and refers to the perception that a given innovation is agreeable, palatable, or satisfactory (Proctor et al., 2011). Fidelity is defined as “the extent to which specified program components are delivered as prescribed” in the original protocol or by the program developers (Berkel et al., 2011). Comprehensive assessment of fidelity provides critical information to inform implementation and dissemination efforts and to address research-to-practice gaps (Breitenstein et al., 2010). Thus, fidelity is an important antecedent for intervention/program effectiveness (Miller & Rollnick, 2014), yet fidelity measures are rarely analyzed together with program outcomes (Schoenwald & Garland, 2013).

To date, only a single overdose prevention program—the manualized iBook opioid overdose prevention program—reported a fidelity measure using the Opioid Overdose Prevention Program Fidelity Checklist (completed by independent observers) with established content validity. Evaluation results from 12 iBook group sessions indicate that the intervention was delivered with a high level of fidelity, as indicated by adhering to the core components of the iBook intervention 97% of the time (Clark et al., 2016). While these results yield important first insights into the usability of one specific manualized OEND training model, there is a paucity of research exploring the relationships among OEND implementation outcomes and intervention outcomes (Breitenstein et al., 2010).

### Study Aims

The current study aimed to evaluate the fidelity of OEND implementation in New York City (NYC). Specifically, we sought to explore the relationships between implementation outcomes (fidelity and acceptability of OEND) and OEND outcomes (overdose recognition and response knowledge and confidence in managing an overdose situation). For the current study, we used Proctor et al.’s (2011) working taxonomy of implementation outcomes to organize key variables and frame our research questions (Proctor et al., 2011). This taxonomy proposes eight distinct implementation outcomes, including the level of analysis (from the individual provider/consumer to the organization/setting) and stage within the implementation process at which the outcome may be most salient (i.e., pre-adoption to late stages of implementation), with the aim to advance their conceptualization and facilitate their operationalization. Implementation outcomes of interest in this study were chosen based on the current stage of implementation and level of analysis. By now, OEND has been widely adopted throughout the US and is maintained or institutionalized within a range of service settings (stage of implementation: mid to late). We were particularly interested in the quality of program delivery (fidelity; level of analysis: individual providers) and satisfaction with various aspects of OEND (acceptability; level of analysis: individual providers and recipients). In line with best practices for treatment integrity (Keller-Margulis, 2012), we assessed implementation outcomes from multiple perspectives (OEND trainers, OEND trainees, and an independent observer).

We hypothesized that OEND would improve overdose recognition and response knowledge, and attitudes towards helping in an overdose situation (Doe-Simkins et al., 2009; Jones et al., 2014; McAuley et al., 2010; Piper et al., 2008; Seal, 2005; Strang et al., 2008; Tobin et al., 2009; Wagner et al., 2010; Williams et al., 2014). In addition, we assumed that variations in fidelity are related to variations in intervention outcomes (Trivette & Blase, 2012). We had no a priori hypotheses regarding differential effects of implementation outcomes from different perspectives on intervention outcomes as this is, to our knowledge, the first study to use multi-informant measures of OEND implementation outcome.

## Methods

All study procedures were approved by the New York State Psychiatric Institute Institutional Review Board (IRB protocol #7785).

### Recruitment

Recruitment took place at harm reduction programs, drug-treatment centers, and other official overdose prevention programs identified via the Directory of Registered Opioid Overdose Programs—New York City Region (New York State Department of Health, 2020). NYC is a leader in the implementation of public health programming to prevent death from opioid overdose (New York State Department of Health, 2019a). Naloxone distribution efforts in New York State were formalized in 2007, and the New York State Department of Health set forth local “Guide for New York State’s Registered Opioid Overdose Prevention Programs” for delivering OEND services consistent with New York Public Health Law (New York State Department of Health, 2019b). In 2017, the NYC Department of Health and Mental Hygiene dispensed 61,706 naloxone kits through New York City’s 165 registered overdose prevention programs (National Drug Early Warning System, 2018).

The study team was able to establish contact with 22 opioid overdose prevention programs, and seven programs agreed to participate in the study.

#### Trainers

In NYC, all individuals interested in providing OEND training participate in a 2-h opioid overdose prevention training of trainers provided by the NYC Department of Health and Mental Hygiene to become certified naloxone dispensers (New York City Department of Health and Mental Hygiene, 2021). All program staff are equipped with standardized overdose prevention training slides, brief training script, and training flashcards. However, programs are not required to use any of these training materials; they only need to cover the core components of OEND (see description below) and are free to choose the training method they deem appropriate. Naloxone is provided by the New York State Department of Health to overdose prevention programs at no cost.

OEND trainers at the seven participating overdose prevention programs were approached by a member of the research team. Trainers were staff of the respective program, certified to provide OEND, experienced in OEND training delivery, and at least 18 years of age. Trainers were asked to complete a survey (described in the Survey Assessments section) following the training. Completion took between 2 and 5 min in addition to the training, and no compensation was provided (trainers completed the surveys during their working hours, as agreed upon by program directors). Note that we did not record trainers’ demographic information to provide added protection from identification. In addition, the impact of specific trainer characteristics on training effectiveness was not a primary focus of this study, notwithstanding that this may be an interesting research question for future studies.

#### Trainees

Trainers were asked to provide information about the study (via a flyer) to prospective OEND trainees. As per the New York State Department of Health guidelines, all adults interested in becoming trained overdose responders are eligible to receive OEND and were therefore eligible to participate in this study as trainees. Trainees included people who use drugs, their friends and families, other members of their social network/community, and staff and volunteers at agencies providing services to people who use drugs. In addition, trainees had to be at least 18 years of age, able to fluently speak and read English, and provide informed consent (i.e., no current psychotic disorder or other mental/health condition impairing comprehension and completion of assessments). Trainees were asked to complete a survey (described in the Survey Assessments section) before and after the training. Completion took between 15 and 30 min in addition to the training, and they received $10 compensation for their time.

### Survey Assessments

Only trainings that were scheduled or carried out ad hoc (e.g., during a treatment consultation) at the participating programs were observed for the current study. Note that the training itself was not part of the study procedures. The training content was not changed in any way for the purpose of the study as it was our aim to assess OEND implementation outcomes under everyday practice conditions.

Besides trainees’ sociodemographic and drug use characteristics, we assessed their previous history of OEND training and experience with overdoses to account for differences in baseline knowledge of overdose prevention and reversal. In addition, we asked for reasons for participating in OEND training. Implementation variables and intervention outcomes were assessed with the following measures.

#### Implementation Outcomes

##### Fidelity

We assessed two core components of fidelity: The degree to which an intervention is conducted (a) competently (competence), and (b) according to protocol (adherence) (Carroll et al., 2007; Clark et al., 2016; Dusenbury et al., 2005). In the context of this study, the term “protocol” refers to the training content to be addressed as per the New York State Department of Health guidelines (New York State Department of Health, 2019b). Clark et al. (2016) have described the systematic adaptation of the Fidelity Checklist—a valid and reliable tool for measuring fidelity (Breitenstein et al., 2010)—for fidelity assessment of a group-based community overdose prevention program, including an adherence and a competence subscale. The Opioid Overdose Prevention Program Fidelity Checklist was modified for the purposes of the current study to remove reference to the iBook intervention and replace with New York State OEND training (Clark et al., 2016).

##### Adherence

An essential first step in evaluating fidelity is the definition of core components of a program, defined as “the essential functions or principals, and associated elements and intervention activities (…) that are judged necessary to produce desired outcomes” (Blase & Fixsen, 2013). Therefore, the Fidelity Checklist Adherence Subscale was adapted according to the core components of OEND set out in the New York State Department of Health guidelines. These include training on (a) risk factors of overdose: loss of tolerance, mixing drugs, using alone; (b) signs of an overdose: lack of response to sternal rub, shallow/no breathing, bluish lips or nail beds; and (c) actions: call 911, rescue breathing, rescue position, using naloxone. Given that illicitly manufactured fentanyl—a highly efficacious mu-opioid receptor agonist estimated to be 50–100 times more potent than morphine—and its’ more potent analogs have contributed to an evolving and increasingly lethal opioid epidemic in the US (Martinez et al., 2021), we also inquired about the coverage of fentanyl-related information during the training. Representatives from the NYC Department of Health and Mental Hygiene who provide trainings for all OEND trainers in NYC and experienced OEND trainers advised the research team on items that were deemed mandatory for OEND trainings. The final modified Adherence Subscale included 17 dichotomously scored items coded as “yes” for completion or “no” for non-completion (scores range from 0 to 17; see modified Adherence Subscale in the supplementary material). The same version of the scale was used for adherence ratings by trainers, trainees, and the independent observer.

##### Competence

The original Fidelity Checklist Competence Subscale, assessing the trainer’s group facilitation and process skills, was developed specifically for group settings and for independent observers (Breitenstein et al., 2010). Thus, we developed an observer and a trainee version of the Competence Subscale that could be used for group and individual trainings. In both versions, we omitted items that solely pertained to group settings (e.g., “trainer effectively uses group discussion to teach a principles or strategies for overdose prevention”). In addition, we simplified the language of the trainee version (e.g., we avoided using items with double negation). The final trainee version included 14 items with a 3-point scale rating of skill “rarely or never demonstrated,” “sometimes/occasionally demonstrated,” or “consistently demonstrated” (scores range from 14 to 42). The final observer version consisted of nine items, with the same 3-point scale (range 9–27). Both versions of the modified Competence Subscale are available in the supplementary material.

##### Acceptability

Acceptability of OEND by trainers and trainees was measured as an additional implementation outcome, using the Acceptability of Intervention Measure (AIM). The AIM is one of the very few implementation outcome measures with verified validity and reliability (Weiner et al., 2017).

##### Multi-informant Measures of Implementation Outcomes

It has been recommended to measure fidelity from different perspectives as they can have different relations to program outcomes (Schultes et al., 2015). Therefore, we measured implementation outcomes from the perspective of trainers and trainees for all observed trainings. Trainers were asked to self-report their adherence to OEND core components following each training (using the modified Fidelity Checklist Adherence Subscale) and their acceptability of OEND (using the AIM). Only one acceptability measure per trainer was obtained, even if more than one training provided by the same trainer was included in the study. Trainees were asked to rate their trainer’s competence and adherence (modified Fidelity Checklist Adherence Subscale and Competence Subscale—trainee version) and self-report their acceptability of OEND (AIM) following the training. We included a third perspective by means of observer ratings, using the modified Fidelity Checklist Adherence Subscale and Competence Subscale—observer version. To realize training observations, a member of the research team (LB) was present at trainings whenever possible (three trainings that were conducted ad hoc were unobserved). Clark et al. (2016) reported very high agreement between raters using the Fidelity Checklist, with 14 out of 18 items receiving 100% agreement. Therefore, we deemed it justified to only include personal observations by one rater. This rater was the same for all observations.

#### Intervention Outcomes

Training outcomes were measured by means of changes in trainees’ opioid overdose knowledge and self-perceived ability to manage an overdose from pre- to post-training. Trainees were asked to complete the Opioid Overdose Knowledge Scale (henceforth referred to as the knowledge scale) and the Opioid Overdose Attitudes Scale (henceforth referred to as the attitudes scale) before training (baseline) and immediately after trainings. The knowledge scale (total score range: 0 to 46) is a validated questionnaire to assess the level of knowledge of opioid overdose management, including risk factors of overdose, signs of an opioid overdose, actions to be taken in an overdose situation, naloxone effects, and administration, adverse effects and aftercare procedures (Williams et al., 2013), which are also defined as the core components of OEND (New York State Department of Health, 2019b). The knowledge scale is robust over time, and the scale has proven to have face, content, and construct validity. The validated attitudes scale (total score range 29 to 145) assessed self-perceived ability to manage an overdose, concerns about dealing with an overdose, and willingness to intervene in an overdose situation (Williams et al., 2013).

### Statistical Analysis

Training and trainee characteristics as well as intervention and implementation outcomes are presented descriptively. Given that our data represent trainees (Level 1 unit) who are nested in trainings (Level 2 unit), we first determined whether there is evidence of substantial clustering with respect to the dependent variables (training outcomes: changes in overdose knowledge and attitudes from pre- to post-training) in a variance component model (null/no predictor model) (Heck et al., 2013). Clustered data arise where observations were collected from different groups (e.g., training groups), referred to as clusters. The key feature of clustered data is that observations within a cluster are “more alike” than observations from different clusters (Galbraith et al., 2010). The null or “empty” model contains just one fixed term—the mean—and the variance at each level. In the context of this study, this corresponds to the overall outcome (changes in overdose knowledge and attitudes) in a typical training group, between training group variation and within training group variation. This allows for the calculation of an Intraclass Correlation Coefficient (ICC). In the context of this study, the ICC provides the proportion of the total variation at the training level and indicates how similar individuals within a training group are on the outcome. In addition, we estimated the design effect (*d*_eff_) as a function of the ICC and average cluster size (*c*): *d*_eff_ = 1 + (*c* − 1) × ICC (Muthen & Satorra, 1995). *d*_eff_ measures the expected effect of the design structure (such as correlations among clusters of observations) on the variance of the estimator of interest (e.g., the mean outcome).

For changes in overdose knowledge as the Level 1 outcome and training as the Level 2 unit, the variance of intercepts/means (i.e., how much groups differ from each other) across Level 2 units was not significantly different from 0, *σ*_*μ*0*j*_^2^ = 4.27, Wald *Z* = 1.03, *p* = 0.153, with *d*_eff_ = 1 + (3.93 − 1) × 0.14 = 1.41. Similarly, the variance of intercepts/means across Level 2 units was not significantly different from 0 when using changes in attitudes as the Level 1 outcome, *σ*_*μ*0*j*_^2^ = 0.08, Wald *Z* = 1.18, *p* = 0.119, with *d*_eff_ = 1 + (3.77 − 1) × 0.25 = 1.69. Given that we were primarily interested in the effects of the Level 1 predictors, we applied the rule of thumb that “if the design effect is smaller than two, the effect of clustering can be ignored” (Hox & Maas, 2002). Based on these results, we carried out subsequent analyses using a single-level approach. “Single-level” means that the analysis is carried out at one analytical level—in this case, the individual trainee level.

Stepwise multiple ordinary least squares regressions were calculated to predict changes in opioid overdose knowledge (sum score) and attitudes (mean score) based on OEND implementation outcomes (adherence of the trainer to the protocol, competence of the trainer, acceptability of OEND). Regression analysis is used to provide an estimation of the relationship between a dependent variable and one or more independent or outcome variables. We used the knowledge scale sum score given that non-response to an item on this scale was not counted as missing but as a correct or incorrect response (the instruction for each question on the scale is to tick all answering options that apply). In contrast, attitudes scale responses are recorded on a Likert scale from 1 = completely disagree to 5 = completely agree, which is why we used attitudes scale mean scores as the outcome variable. We modeled each perspective (trainee, trainer, and observer) in a separate regression model. Analyses were performed using the Statistical Product and Service Solutions (SPSS®) version 18 software platform, which is commonly used to in the social sciences to conduct statistical analyses.

## Results

### Training Characteristics

Sixteen OEND trainings, conducted in-person between April 2019 and March 2020, were evaluated. These 16 trainings were delivered by 10 different trainers. One trainer delivered six trainings and another trainer two; for all remaining trainers only one training was observed. Ten were individual trainings and six were group trainings (mean # trainees = 4.7, range 4 to 29). Fourteen trainings were delivered at the program site, one was delivered at a community health center, and one at a veteran’s housing site. Training duration ranged from 7 to 70 min (*M* = 38.65, SD = 25.34). Individual trainings were significantly shorter (on average 13.29 min, SD = 6.10) than group trainings (on average 42.55 min, SD = 24.96), *t*(70) = − 3.07, *p* = 0.003.

### Trainee Characteristics

Eighty-two trainees participated in the 16 trainings evaluated in this study. One trainee was excluded from study participation because they were younger than 18 years, and five trainees declined to participate. Seventy-six trainees were enrolled in the study; one assessment was excluded from data analysis because less than 5% of the survey were completed. Table 1 displays trainees’ sociodemographic and drug use characteristics, and their previous experience with overdoses and overdose prevention training. Trainees had a mean age of 39.39 (SD = 13.55) and the majority had drug use experience (other than cigarettes). Of those who reported having ever used heroin (29 participants), 12 (41.4%) were current users (Table 1). The 12 participants who had overdosed in the past had experienced between 1 and 9 overdoses, and overdoses had been experienced between 6 months and 26 years prior to assessment. More than one third of participants (38.7%) had witnessed between 1 and 10 overdoses, between 1 month and 20 years prior to assessment. Of those who reported that they had received OEND training before, four trainees had received it within the past year, and three within the past 2 years.

**Table 1:** Trainee sociodemographic and drug use characteristics, and overdose and overdose prevention training experience (*N* = 75)

Characteristic	*n*	*%*
Sex		
Female	24	32.0
Male	41	54.7
Transgender	4	5.3
Race/ethnicity		
Black/African American	25	33.3
Latino/a or Hispanic	16	21.3
White/Caucasian	16	21.3
Asian	4	5.3
More than one race	11	14.7
Drug use		
Ever used drugs (other than cigarettes)	65	86.7
Ever used heroin	29	38.7
Currently receiving treatment for drug use	28	37.3
Overdose and overdose prevention experience		
Ever experienced an overdose	12	16.0
Ever witnessed an overdose	29	38.7
Ever received a naloxone kit	25	33.3
Ever received overdose prevention training	21	28.0

*OEND* Overdose Education and Naloxone Distribution, *OOKS* Opioid Overdose Knowledge Scale, *OOPP* Opioid Overdose Attitudes Scale

The most frequently cited reasons for participating in OEND training were that trainees might witness an overdose in their community and a general interest in overdose prevention (Table 2). More than one third of trainees were referred to OEND training by a health professional but only 12% saw themselves at risk of an overdose. Twenty percent of trainees took the training because they might encounter an overdose at their workplace, and others reported that their friends, family members, and/or partners were at risk of experiencing an overdose.

**Table 2:** Trainees’ reasons for participating in Overdose Prevention Training

Reason	*n*	%
A counselor/doctor/health professional suggested it to me	28	37.3
I asked to receive overdose prevention training	26	34.7
I asked to receive naloxone	23	30.7
A friend of mine is at risk of experiencing an overdose	16	21.3
I am at risk of experiencing an overdose	9	12.0
A family member of mine is at risk of experiencing an overdose	7	9.3
My partner/spouse is at risk of experiencing an overdose	5	6.7

Multiple answers possible

### Intervention and Implementation Outcomes

Trainees’ overdose knowledge significantly increased from an average sum score of 27.72 (SD = 7.74) pre-training to 33.12 (SD = 7.70) post-training, *t*(71) = − 8.12, *p* < 0.001. In addition, trainees’ overdose attitudes significantly improved from a mean score of 3.53 (SD = 0.50) pre-training to 4.01 (SD = 0.55) post-training, *t*(65) = − 6.85, *p* < 0.001. Changes in overdose knowledge were significantly correlated with changes in overdose attitudes, *r* = 0.57, *p* < 0.001. There were no baseline (pre-training) differences in knowledge scale sum score depending on trainees’ experience with overdoses as a witness, *t*(68) = 0.19, *p* = 0.850, or overdose victim, *t*(68) = 0.86, *p* = 0.395, or heroin use experience (current and past use combined), *t*(72) = 0.59, *p* = 0.559. Likewise, there were no baseline (pre-training) differences in attitudes scale mean score depending on experience with overdoses as a witness, *t*(67) = 0.13, *p* = 0.898, or overdose victim, *t*(67) = 1.07, *p* = 0.288, or heroin use experience, *t*(71) = − 0.26, *p* = 0.799. However, trainees who had received previous overdose prevention training had higher pre-training knowledge scale sum scores, *t*(65) = 2.06, *p* = 0.045, and attitudes scale mean scores, *t*(64) = 3.11, *p* = 0.003.

Table 3 displays implementation outcomes from the perspective of trainees, trainers, and the independent observer, and the relationships between these variables (bivariate correlations). Trainees’ adherence and acceptability ratings both correlated medium-to-high with trainers’ self-ratings. In addition, trainees’ adherence rating was associated with their acceptability of the intervention. However, trainees’ ratings of adherence and competence did not correlate with observer ratings of these variables.

**Table 3:** Means, standard deviations, and correlations of implementation outcomes

Implementation outcome (perspective)	*M*	SD	1	2	3	4	5	6	7
1. FC—Adherence of the trainer to the protocol (trainee)	16.04	2.64	1	0.43*** [0.21, 0.61]	0.22 [− 0.02, 0.43]	0.11 [− 0.13, 0.34]	0.29* [0.05, 0.49]	0.57*** [0.39, 0.71]	0.40** [0.18, 0.58]
2. FC—Adherence of the trainer to the protocol (trainer)	16.17	1.20	–	1	0.42*** [0.21, 0.60]	0.06 [− 0.18, 30]	0.49*** [0.28, 0.65]	0.35** [0.12, 0.54]	0.80*** [0.69, 0.87]
3. FC—Adherence of the trainer to the protocol (observer)	14.46	2.84	–	–	1	− 0.12 [− 0.35, 0.13]	0.81*** [0.71, 0.88]	0.24* [0.01, 0.46]	0.06 [− 0.18, 0.29]
4. FC—Competence of the trainer (trainee)	33.38	7.79	–	–	–	1	0.13 [− 0.12, 0.35]	0.04 [− 0.20, 0.27]	0.00 [− 0.24, 0.24]
5. FC—Competence of the trainer (observer)	20.92	3.99	–	–	–	–	1	0.23 [− 0.01, 0.45]	0.19 [− 0.06, 0.41]
6. AIM—Acceptability of OEND (trainee)	17.66	2.69	–	–	–	–	–	1	0.35** [0.12, 0.54]
7. AIM—Acceptability of OEND (trainer)	17.96	2.12	–	–	–	–	–	–	1

Bracketed numbers represent 95% confidence intervals [lower bound, upper bound]

*AIM* Acceptability of Intervention Measure; *FC* Fidelity Checklist

*p < 0.05. **p < 0.01. ***p < 0.001

Stepwise multiple regression models were calculated to predict intervention outcomes [changes in opioid overdose knowledge (sum score) and attitudes (mean score) from pre- to post-training] based on implementation outcomes (adherence of the trainer to the protocol, competence of the trainer, and acceptability of OEND) from the perspectives of trainees, trainers, and an independent observer. We included trainees’ overdose prevention training experience (yes/no) as an additional predictor, given the baseline differences in overdose knowledge and attitudes between those who did and those who did not have previous training experience (see above). In addition, length of training (in minutes) was included as a predictor in the model as there was a significant correlation between length of training and change in overdose knowledge, *r* = 0.33, *p* = 0.004, and attitudes, *r* = 0.40, *p* = 0.001.

Table 4 presents the coefficient estimates of the final regression models by perspective (trainee, trainer, and observer). For the trainee perspective models, only duration of training added significantly to the prediction of change in knowledge scale sum score, *F*(1, 69) = 8.56, *p* = 0.005, and attitudes scale mean score, *F*(1, 63) = 12.28, *p* = 0.001. No other predictors entered into the equation at subsequent steps of the analyses. Participants’ knowledge scale sum score improved by 0.07 points and mean attitudes scale mean score improved by 0.01 points for each additional training minute (Table 4).

**Table 4:** Results of the final models from the stepwise multiple linear regression analyses

	*B*	SE	*p*	*R*^2^		*B*	SE	*p*	*R*^2^
Trainee perspective									
OOKS change^a^				0.110	OOAS change^c^				0.163
Training length^b^	0.07	0.03	0.005		Training length^b^	0.01	0.00	0.001	
Trainer perspective									
OOKS change^a^				0.159	OOAS change^c^				0.214
Training length^b^	0.06	0.03	0.021		Training length^b^	0.01	0.00	0.005	
Training experience	2.44	1.22	0.049		Training Experience	0.25	0.12	0.048	
Observer perspective									
OOKS change^a^				0.128	OOAS change^c^				0.163
Trainer Adherence^d^	0.69	0.22	0.003		Trainer Adherence^e^	–	–	–	
Training length^b^	–	–	–		Training length^b^	0.01	0.00	0.001	

*B* unstandardized regression coefficient; *SE* standard error

^a^Change in Opioid Overdose Knowledge Scale (OOKS) sum scores from pre- to post-training

^b^Length of the training session in minute

^c^Change in Opioid Overdose Attitudes Scale (OOAS) mean scores from pre- to post-training

^d^Trainees’ previous experience with training (have/have not received OEND before

^e^Adherence of the trainer to the protocol rated by the independent observer

For the trainee perspective models, duration of training added significantly to the prediction of change in knowledge scale sum score, *F*(1, 70) = 8.68, *p* = 0.004, and attitudes scale mean score, *F*(1, 64) = 12.47, *p* = 0.001. At step 2 of the analyses, previous training experience entered into the equation for predicting knowledge scale sum score change, *F*(2, 69) = 6.53, *p* = 0.003, and attitudes scale mean score change, *F*(1, 63) = 8.56, *p* = 0.001. Participants without previous training experience had greater increases from pre- to post-training in knowledge scale sum scores and attitudes scale mean scores (Table 4).

Adherence of the trainer to the protocol rated from the observer perspective added significantly to the prediction of change in knowledge scale sum score, *F*(1, 67) = 9.81, *p* = 0.003, and explained 13% of the variance in outcome. Trainees’ knowledge scale sum scores improved by 0.69 points for each additional action taken by the trainer (i.e., each “yes” on the adherence subscale as rated by the observer; Table 4). For the prediction of attitudes scale mean score change from the observer perspective, only duration of training added significantly to the model, *F*(1, 61) = 11.89, *p* = 0.001, and no other predictors entered into the equation at subsequent steps of the analyses.

## Discussion

OEND is increasingly accepted as an effective public health intervention to reduce overdose fatalities. Nonetheless, we know little about program implementation outcomes, specifically the fidelity of training delivery, and how these may impact intervention outcomes such as overdose knowledge and attitudes. The current study addressed this question by evaluating implementation outcomes of OEND from multiple perspectives and analyzing them in relation to intervention outcomes.

It has been previously reported that overdose education varies greatly within and across overdose prevention programs (Clark et al., 2014). Our findings indicate a similarly large variation in OEND trainings in NYC in terms of training lengths, training group size, and training audience (i.e., varying experience with opioids use, witnessing and experiencing overdoses, and previous overdose prevention training). There were no differences in trainees’ pre-training knowledge about or attitudes towards overdose prevention dependent on experience with heroin use or previous exposure to opioid overdoses, indicating that training mixed groups of community members likely to witness an overdose event and opioid users at risk of experiencing an overdose is feasible and reasonable.

Our *intervention* outcome results are in line with numerous studies indicating that OEND training increases participants’ knowledge about and confidence in overdose management (Doe-Simkins et al., 2009; Jones et al., 2014; McAuley et al., 2010; Piper et al., 2008; Seal, 2005; Strang et al., 2008; Tobin et al., 2009; Wagner et al., 2010; Williams et al., 2014). Our *implementation* outcomes indicated that, overall, trainers delivered the intervention competently and as prescribed (i.e., their adherence was rated high from all perspectives). In addition, both trainers and trainees found OEND highly acceptable (i.e., the intervention met their approval).

To the best of our knowledge, this is the first study to utilize multi-informant measures of OEND implementation outcomes. Previous studies of other interventions measured fidelity from different perspectives and showed that using different sources for fidelity measures leads to different results (Ennett et al., 2011; Lillehoj et al., 2004; Schultes et al., 2015). Likewise, our results indicate that OEND implementation outcomes differed depending on the role of the fidelity rater in relation to the intervention (provider, recipient, independent). Specifically, the mean adherence score assessed from the observer perspective was lower than trainer’s self-ratings and trainee ratings and showed greater variability. The observer in this study had previous observation and data collection experience with regard to evaluating overdose prevention trainings, reviewed the Fidelity Checklist manual (Clark et al., 2016) in detail before the training observations, and was not actively participating in the training. Thus, the observer may have been able to appraise the trainers more critically and perhaps evaluate more accurately which elements had or had not been covered in trainings. Nonetheless, it cannot be ruled out that observer ratings may be subjective or biased (Hoyt & Kerns, 1999), albeit any bias would have likely been consistent across ratees given that all observations were conducted by the same observer. In addition, it is possible that social desirability bias led to more favorable trainee ratings—even though all trainees were informed that trainers would not be notified of their ratings—and trainer self-ratings (Lillehoj et al., 2004; Mowbray et al., 2003).

Fidelity rated from different perspectives contributed differently to the prediction of outcomes. Trainers’ adherence to the protocol rated by the observer contributed to the prediction of overdose knowledge outcomes. However, implementation outcomes from the perspective of trainers and trainees did not significantly predict intervention outcomes. These results indicate that an independent perspective might be the most useful to evaluate OEND training fidelity. Nonetheless, assessments from the perspective of individuals who have an active role in an intervention could be useful in other ways. For example, the trainer version of the Adherence Subscale may help cue trainers to the training guidelines and serve as a self-monitoring tool (Clark et al., 2016).

In addition to adherence measured from the observer perspective, training lengths predicted overdose knowledge and attitudes, with longer training sessions resulting in greater changes from pre- to post-training. Longer trainings may cover additional details and/or repeat key information to increase memory consolidation and trainees’ confidence in applying their knowledge in an overdose situation. However, this result should not be taken as advocacy for extending the training length indefinitely. The longest training session observed for the current study lasted 70 min, and trainings that exceed this time frame may negatively impact trainees’ acceptability of OEND and willingness to participate in this important harm reduction measure. Oftentimes, it is not practical to provide extended training sessions (e.g., when training is provided in the context of a treatment visit), and brief trainings (less than 20 min) have shown to be sufficient to impart basic overdose knowledge (Behar et al., 2015; Jones et al., 2014). In addition, we found that individuals who had received previous OEND training had higher pre-training knowledge and more confidence in overdose management, but they had less gain in these outcomes through the training. Nonetheless, periodic re-trainings—recommended by the New York State Department of Health guidelines every 2 years—may serve to refresh trainees’ memory and provide updates on any new developments/findings in the context of overdose prevention.

Of note, it is largely unknown how well self-report measures of overdose knowledge and attitudes predict actual behavior in an overdose situation (Franklin Edwards et al., 2020). Because direct observations of laypersons behavior in real overdose situations are generally not feasible, due to the unpredictable nature of overdoses, some researchers have attempted to measure overdose response proficiency (i.e., how well laypersons are able to translate what they have learned during training into behavior) in a simulated overdose situation. These studies indicate variations in the ability of trained laypersons to administer naloxone (Eggleston et al., 2018, 2020). While our study and others have shown that commonly used OEND practices lead to improved opioid overdose knowledge and attitudes toward overdose management (Clark et al., 2014; European Monitoring Centre for Drugs & Drug Addiction, 2015; McDonald & Strang, 2016), continued research addressing the gap between intended or anticipated behavior and actual behavior is required to clarify the real-world implications of these outcomes.

### Limitations

Several limitations of our study should be noted when interpreting our findings. First, our sample was small. We had planned to include up to 200 trainees in this study; however, data collection was interrupted by the COVID-19 pandemic, which led to the suspension of non-essential research at New York State Psychiatric Institute in March of 2020. In addition, protective measures against the spread of COVID-19 included suspension of in-person OEND trainings. Since then, many overdose prevention programs implemented adaptations to their OEND strategy (Courser & Raffle, 2021). For example, they organized “Drive-Thru” events, provided training over the phone or through videos posted on the agency’s website, and mailed naloxone kits to individuals who had taken an online training. While these are innovative and successful responses to a rapidly emerging need, we decided to close data collection for the current study as these adaptions are (a) not comparable with the previous in-person training format, and (b) no protocol or guideline against which fidelity could be measured has been developed yet for these new formats. Future research will be tasked with modifying the Fidelity Checklist for use in these emerging contexts.

A second limitation involves the use of a convenience sampling approach. Even though our sample was diverse in terms of age, sex, and race/ethnicity, it is not known whether it is representative of the larger OEND trainee population. A third limitation is that our average cluster size was less than 10 and use of single-level models may have resulted in biased standard errors for the Level 1 (trainee level) regression coefficient (Lai & Kwok, 2015). The regression coefficient is a statistical measure which indicates the change in the value of the dependent variable (intervention outcomes) corresponding to the unit change in the independent variable (implementation outcomes). Future studies with larger samples should explore coefficient variations with multilevel techniques (i.e., approaches that can handle clustered or grouped data).

A fourth limitation is that the very high trainer and trainee ratings of OEND acceptability may be subject to selection bias. Due to the naturalistic design of our study, we only included trainees who had already agreed to receive OEND training and staff members who were certified OEND trainers. The acceptability of OEND may be lower among those who are not actively taking part in OEND, either because they refuse to do so or because they have not been offered to receive/provide OEND. In addition, studies that intend to measure fidelity from different perspective may consider interviewing trainers (or other raters) to ascertain whether they understand the fidelity questions in the same way as the independent observer(s). Interviews may also be useful for further exploring the impact of training context and group size on opportunities to adapt/tailor the training content and materials or facilitate problem solving.

A fifth limitation is that our results are specific to OEND delivered in NYC. NYC provides an ideal context to study OEND implementation as this harm reduction measure is widespread (at the time of study initiation, 128 registered overdose prevention programs were identified in the NYC area), training of trainers is standardized (all OEND trainers participate in a training of trainers provided by the NYC Department of Health and Mental Hygiene), and a local training protocol as well as standardized training materials are available (New York City Department of Health and Mental Hygiene, 2021). Future studies are tasked with studying OEND implementation outcomes in other contexts, such as rural areas where OEND and general harm reduction information and offers may be less available, to examine needs and barriers that arise with OEND adoption. Particularly in areas where OEND is less readily available than in NYC, studying factors that facilitate the adoption and/or penetration of OEND, in addition to other implementation outcomes such as appropriateness and feasibility [salient prior to or early in the adoption stage (Proctor et al., 2011)], will further advance our understanding of implementation processes. A sixth limitation is that, given our focus on fidelity and acceptability, we were not able to explore potential interrelations of these with other implementation outcomes. Future empirical work should explore the dynamic and complex interrelations between implementation outcomes at different stages in the implementation process (Lewis et al., 2015).

## Conclusions

There is an emphasis on developing new pharmacological and behavioral interventions for the treatment of opioid use disorder and opioid overdose (Volkow & Blanco, 2021). However, systematically examining the context and fidelity of community-level implementation of evidence-based interventions is equally important to increase the adoption of these practices and help attenuate the prevailing opioid overdose epidemic (Knudsen et al., 2020). Our findings may help advance understanding of how to best evaluate implementation outcomes of harm reduction measures as they suggest differences depending on the evaluator's perspective. Specifically, they indicate that fidelity should be measured from an independent perspective (i.e., an individual who is experienced with fidelity rating but not directly involved in the intervention). Additional research may be required to fully understand how assessments from the perspectives of intervention providers and recipients can contribute to evaluate intervention delivery or participant outcomes.

## Supplementary Information

Below is the link to the electronic supplementary material.

Supplementary file1 (PDF 167 KB)
