# Supplementary material for: Multi-informant Implementation and Intervention Outcomes of Opioid Overdose Education and Naloxone Distribution in New York City
Source: Glob Implement Res Appl. 2021 Sep 20;1(3):209–22. doi: 10.1007/s43477-021-00021-4 (PMC8450721; doi:10.1007/s43477-021-00021-4)
Supplement: Supplementary file 1 — Supplementary file1 (PDF 167 KB) [file 43477_2021_21_MOESM1_ESM.pdf]

# <u>Supplementary Material</u>

## Modified OOPP Fidelity Checklist – Adherence Subscale

**Please answer yes or no whether <u>the trainer</u> [for trainer assessment replace with "you"] performed the following actions:**

**The trainer [for trainer assessment replace with "I"] …**

| | YES | NO |
|---|---|---|
| … defined opioids (provided examples of prescription and illicit opioids). | | |
| … explained what naloxone (Narcan®) does. | | |
| … talked about fentanyl. | | |
| … explained that naloxone (Narcan®) works on fentanyl. | | |
| … discussed risk factors for opioid overdose. | | |
| … explained how to recognize an overdose. | | |
| … demonstrated how to check for responsiveness (sternal rub). | | |
| … instructed to call 911 if the sternal rub does not wake the person up. | | |
| … discussed reasons for contacting 911. | | |
| … explained the Good Samaritan Law. | | |
| … demonstrated how to use naloxone (Narcan®). | | |
| … explained what to do if the person who overdosed becomes responsive (aftercare). | | |
| … introduced rescue breathing. | | |
| … introduced the recovery position. | | |
| … explained how to report naloxone use/overdose reversal. | | |
| … explained how to get a refill after use, loss or expiration of naloxone (Narcan®). | | |
| … made sure there were no unanswered questions. | | |

**Modified OOPP Fidelity Checklist – Competence Subscale – Trainee Version**

**Please rate the following statements relating to the opioid overdose prevention training you just participated in. If a statement is not applicable to the training situation you experienced, please check (✓) "Does not apply".**

| | Rarely or never | Sometimes/ occasionally | Consistently | Does not apply |
|---|---|---|---|---|
| I felt heard and understood by the trainer. | O | O | O | O |
| The trainer was polite and considerate regarding my feelings and comments. | O | O | O | O |
| The trainer made judgements or stated personal opinions regarding my statements, feelings, or thoughts. | O | O | O | O |
| The trainer responded to my opinions and ideas in a supportive manner. | O | O | O | O |
| The trainer summarized important points. | O | O | O | O |
| The trainer clearly responded to my questions and comments. | O | O | O | O |
| The trainer made sure that uncertainties I had regarding opioid overdose prevention were clarified. | O | O | O | O |
| If issues came up (e.g., unsure how to react in a particular overdose situation), the trainer helped me to come up with a plan to address or solve them. | O | O | O | O |
| The trainer was open to my thoughts and feelings. | O | O | O | O |
| I felt that the training was a safe place to share thoughts and feelings. | O | O | O | O |
| The trainer imposed his/her ideas on me. | O | O | O | O |
| If I disagreed with something that was said during the training, the trainer engaged in a discussion with me. | O | O | O | O |
| The trainer addressed potential challenges that may come up in an overdose situation. | O | O | O | O |
| My trainer and I talked about how to use my new skills as an overdose responder to address these challenges. | O | O | O | O |

**Modified OOPP Fidelity Checklist – Competence Subscale – Observer Version**

**Please rate the trainer on the following scale:**

**1= skill rarely or never demonstrated (skill demonstrated <25% of the time)**

**2= skill sometimes/occasionally demonstrated (skill demonstrated 25-75% of the time)**

**3= skill consistently demonstrated (skill demonstrated >75% of the time)**

**If a statement is not applicable to the specific training situation, please check (✓) "Does not apply".**

| | **Rarely or never demonstrated** | **Sometimes/ occasionally demonstrated** | **Consistently demonstrated** | **Does not apply** |
|---|---|---|---|---|
| Actively listens when trainee is talking. | ① | ② | ③ | |
| Communicates with trainee in a respectful, positive, and non-judgmental manner. | ① | ② | ③ | |
| Appropriately reinforces trainee's ideas and opinions. | ① | ② | ③ | |
| Correctly conveys/communicates components of opioid overdose prevention. | ① | ② | ③ | |
| Facilitates problem solving. | ① | ② | ③ | |
| Facilitates sharing of trainee's ideas (or in case of group training among trainees). | ① | ② | ③ | |
| Does not impose his/her ideas on trainee. | ① | ② | ③ | |
| Effectively responds when trainee is resistant to new strategies or ideas. | ① | ② | ③ | |
| Helps trainee anticipate challenges using the new skills. | ① | ② | ③ | |
